# Clinical Profile of Pediatric Neurology Disorders: A Study From a Semi-Urban Medical College in Northwestern India

**DOI:** 10.7759/cureus.30359

**Published:** 2022-10-16

**Authors:** Girish Kumar, Vandana Sharma, Amit Kumar

**Affiliations:** 1 Pediatric Medicine, Dr. Radhakrishnan Government Medical College Hamirpur, Hamirpur, IND; 2 Anatomical Sciences, Dr. Radhakrishnan Government Medical College Hamirpur, Hamirpur, IND

**Keywords:** cerebral palsy, developmental delay, seizures, children, disorders, neurological

## Abstract

Introduction: Neurological disorders are characterized by dysfunction in any part of the nervous system and are a major cause of disability among children and adolescents* *in developing countries, just as it is in India. There is a lack of information on the prevalence of neurological disorders in developing countries due to the lack of quality health information and a lack of awareness of these disorders. This local study aims to provide an understanding of the profile and characteristics of neurological disorders in children that will aid in the development and implementation of preventive healthcare strategies.

Methods: A retrospective observational study was conducted in the Department of Pediatrics. All pediatric neurology patients' data were retrieved from January 2020 to December 2020.

Results: Of the 12,782 children seen in the pediatric outpatient department (OPD), 133 (1.04%) had neurological disorders and about 65% were male. Childhood seizures 92 (69%) and developmental delay 13 (9.7%) were the most common neurological conditions, although there was an overlap of the conditions.

Conclusion: This study provides some valuable information about common neurological disorders in children. Seizures, cerebral palsy, and developmental delay were the most common neurological disorders in children.

## Introduction

Neurological disorders are characterized by dysfunction in any part of the nervous system. Factors that contribute to neurological disorders in children include genetic diseases, neurotoxins, hypoxia, infections, and injuries. Neurologic disorders in children manifest as impaired physical, memory, motor, language, and cognitive functioning. The malfunction caused may lead to various chronic problems.

Neurological diseases are a major cause of disability among children and adolescents in developing countries like India [1-2]. These children face a further burden of poverty, inadequate health facilities, and a lack of community services and aftercare [3]. Genetic factors, body abnormalities, and metabolic disorders play an essential role in medical diseases in children. However, in resource-poor countries, lack of obstetric care, poverty, infection, ignorance, insufficient immunization, deficiency diseases, and poor living conditions are considered to be the main risk factors in the etiology of medical neurological disorders [4-6]. There is a lack of information on the prevalence of neurological disorders in developing countries due to a lack of quality health information and awareness of these disorders. This local study aims to provide an understanding of patterns and characteristics of neurological disorders in children that will aid in developing and implementing better healthcare strategies.

## Materials and methods

This is a retrospective observational study conducted in the Department of Pediatrics. The pediatric neurology clinic is held once a week in the pediatrics department. Children with neurological disorders are referred from general pediatric clinics; they also present directly to the clinic. Each patient is subjected to a thorough examination in order to establish a diagnosis. Based on the history and examination, a provisional diagnosis is made, and the patient is entered into the pediatric neurology register. In the register, variables such as age, gender, residence, and reason for referral are recorded. Patients who require an evaluation are investigated and started on the treatment as required. The institutional ethical committee approved the study but did not request an institutional review board (IRB) since this is a retrospective study without any identifying information.

Data from the registry were retrieved for one year, from January 2020 to December 2020; however, patients were not contacted directly in any way. The study included all patients with neurologic disorders between the ages of one month to 18 years. Children under the age of one month and those suffering from other systemic illnesses were excluded from the study.

The data were entered into a Microsoft Excel worksheet. Patients were divided into four age groups: under one year, one year to five years, five to ten years, and over ten years. Age, gender, and neurological diagnosis were all analyzed as percentages and numbers. When a patient had two or more diagnoses, the major one was chosen and the patient was only entered once. In the case of major neurological diagnoses, subgroup analysis was performed.

## Results

The age and gender distribution of subjects are shown in Table 1. In the Department of Pediatrics, 12,782 children were seen in the outpatient department (OPD), 133 (1.04%) of whom had neurological symptoms and were registered in the department's pediatric neurology registry.

**Table 1:** Age and sex-wise distribution of study participants

Parameters	Male N (%)	Female N (%)	Total N (%)
Total Patients	86 (64.7%)	47(35.3)	133 (100%)
Age distribution			
Less than 1 year	5 (3.75%)	5 (3.75%)	10 (7.5%)
1 to 5 years	19(12.3%)	16(12%)	35 (26.3%)
5 to 10 years	38(28.5%)	16(12%)	54(40.6%)
Above 10 years	24(18%)	10(7.55)	34(25.5%)

Table 2 shows the distribution of neurological disorders among children. Childhood seizures and developmental delay were the most common neurological conditions, although there was an overlap of these conditions. Childhood seizures accounted for 92 (69%) cases and developmental delays were 13 (9.7%).

**Table 2:** Clinical spectrum of neurological disorders ADHD: attention-deficit hyperactivity disorder

Sr. No	Parameters	Total
1	Seizures	92(69%)
2	Cerebral Palsy	26(19.5%)
3	Development delay	13(9.7%)
4	Migraine	1(0.7%)
5	ADHD	1(0.7%)
	Total patients	133

In a subgroup analysis of 92 patients with childhood seizures, 86 (84.2%) were idiopathic, and two (1.8%) were refractory seizures. In developmental delay, six (35%) cases were of global developmental delay out of which five were associated with visual impairment and one case had associated seizure (Table 3).

**Table 3:** Sub-group analyses of common neurological conditions ADHD: attention-deficit hyperactivity disorder

Diagnosis	Sub- group (N and %)
Childhood seizures (92)	Seizure disorders 86(84.2%)
	Seizures with visual impairment 3 (2.8%)
	Febrile seizures 1 (0.9%)
	Seizures and pinealoblastoma 1(0.9)
	Refractory seizures 2(1.8%)
Cerebral palsy (26)	Cerebral palsy with speech delay 4(15.3%)
	Cerebral palsy (Motor delay) 10 (38%)
	Spastic cerebral palsy, with Seizure 4((15.3%)
	Cerebral palsy with vision impairment 5(19.2%)
	Cerebral palsy with ADHD 1(3.8%)
	Cerebral palsy, Diplegia 2(7.6%)
Developmental delay 13(9.7%)	Developmental delay with learning disorder 6 (46.2 %)
	Developmental delay with hearing impairment 1 (7.7 %)
	Developmental delay with language delay 1 (7.7 %)
	Developmental delay with seizures 4 (30.8 %)

## Discussion

The global burden of neurological disease is the highest in Asia and sub-Saharan Africa [5]. Neurological disorders account for more than 20% of the world’s disease burden [6-7]. Child neurology and developmental pediatrics are not well established at the primary level in developing countries; primary care centers lack basic diagnostic and treatment facilities for the management of childhood neurological disorders. Male predominance was found to be consistent with earlier studies from India that reported higher prevalence among males as compared to females [8-10]; a similar pattern of male predominance was also found in other parts of the world [11-12].

We found seizure disorders as the most common neurological disorder in children; in our case, it was 92 (69%) patients. This is similar to the study by Singhi who reported that epilepsy constitutes 85% of pediatric neurology OPD patients [13]. Other studies by Burton et al. and Banoo et al. reported seizure disorder as a common neurological problem in 57% and 42.7%, respectively [7-8].

A significant proportion of these patients had cerebral palsy and other epilepsy syndromes. The high percentage of seizure disorders may be due to increased awareness that these are medical conditions that can be treated. Cerebral palsy in 26 (19.5%) patients was the second most common neurological disorder in our study which is similar to other studies [8,11,14-15]. Many of the affected children require specialized care and rehabilitative services and multidisciplinary teamwork.

Significant improvements in neonatal care services have resulted in increased survival of very low birth weight and premature babies. Both perinatal asphyxia and prematurity constitute major risk factors for cerebral palsy. Despite the high incidence of cerebral palsy in our center, no facilities exist for the adequate care of these patients. In our study, 13 (9.7%) patients were found to have a developmental delay as their primary neurological issue. According to US studies, 16%-18% of children under the age of 18 have behavioral or developmental problems [16-17]. However, research on children under the age of two shows that India has a 1.5%-2.5% rate of developmental delay [18-19]. One patient was found to have a migraine and one had attention-deficit hyperactivity disorder (ADHD).

The study had some limitations; it was a retrospective, hospital-based study with a small number of cases.

## Conclusions

Despite the study's limitations, the current study provides some valuable information on common neurological disorders in children. Seizures, cerebral palsy, and developmental delay were the most common neurological disorders in children.
